# An ultra-dense library resource for rapid deconvolution of mutations that cause phenotypes in *Escherichia coli*

**DOI:** 10.1093/nar/gkv1131

**Published:** 2015-11-17

**Authors:** Ralf B. Nehring, Franklin Gu, Hsin-Yu Lin, Janet L. Gibson, Martin J. Blythe, Ray Wilson, María Angélica Bravo Núñez, P. J. Hastings, Edward J. Louis, Ryan L. Frisch, James C. Hu, Susan M. Rosenberg

**Affiliations:** 1 Department of Molecular and Human Genetics, Baylor College of Medicine, Houston, TX 77030, USA; 2 Department of Biochemistry and Molecular Biology, Baylor College of Medicine, Houston, TX 77030, USA; 3 Department of Molecular Virology and Microbiology, Baylor College of Medicine, Houston, TX 77030, USA; 4 The Dan L. Duncan Cancer Center, Baylor College of Medicine, Houston, TX 77030, USA; 5 Deep Seq. Centre for Genetics and Genomics, Queens Medical Centre, University of Nottingham, Nottingham NG7 2UH, UK; 6 Undergraduate Program in Genomic Sciences, National Autonomous University of Mexico, 62210 Cuernavaca, Mexico; 7 Department of Biochemistry and Biophysics, Texas A&M University and Texas Agrilife Research, College Station, TX 77843, USA

## Abstract

With the wide availability of whole-genome sequencing (WGS), genetic mapping has become the rate-limiting step, inhibiting unbiased forward genetics in even the most tractable model organisms. We introduce a rapid deconvolution resource and method for untagged causative mutations after mutagenesis, screens, and WGS in *Escherichia coli*. We created Deconvoluter—ordered libraries with selectable insertions every 50 kb in the *E. coli* genome. The Deconvoluter method uses these for replacement of untagged mutations in the genome using a phage-P1-based gene-replacement strategy. We validate the Deconvoluter resource by deconvolution of 17 of 17 phenotype-altering mutations from a screen of *N*-ethyl-*N*-nitrosourea-induced mutants. The Deconvoluter resource permits rapid unbiased screens and gene/function identification and will enable exploration of functions of essential genes and undiscovered genes/sites/alleles not represented in existing deletion collections. This resource for unbiased forward-genetic screens with mapping-by-sequencing (‘forward genomics’) demonstrates a strategy that could similarly enable rapid screens in many other microbes.

## INTRODUCTION

The evolution of life from common ancestors has important consequences for biology. Because fundamental biochemical reactions are conserved, discoveries are often transferable across the tree of life. Simple organisms can catapult general advances in cell and molecular biology. Perhaps the most well studied species is the bacterium *Escherichia coli*, which has been favored historically by geneticists, physicists turning to biology, and others because of its relative simplicity and the ease of propagation in the laboratory [reviewed by (1)]. The depth of understanding of and facility of methods for *E. coli* have pushed its recruitment for production of biofuels (2), primary metabolites (3), insulin (4) and tools for engineering other organisms [e.g. (5,6)]. *Escherichia coli* was used to observe single molecules in living cells with millisecond time resolution and nanometer spatial precision (7) and for the recording of intermittent mRNA production (8), and its viruses for engineering of genetic, molecular, and cell biological tools for all organisms [e.g. (9,10)]. Most of our understanding of DNA replication, gene expression and protein synthesis originated from studies in *E. coli*, as did discovery and elucidation of conserved mechanisms of DNA mismatch repair, base- and nucleotide–excision repair, DNA-damage response, repair of DNA double-strand-breaks and homologous-recombination, central in human cancers (11).

Because of their rapid growth rates and ease and precision of genetic manipulation, microbial models have led molecular-biological discovery in forward-genetic screens for unbiased discovery of new gene functions. Unbiased forward genetics uses random, usually chemical mutagenesis to create many kinds of (usually) base-substitution mutations, not biased toward null/knock-out alleles. The random, unbiased mutants are screened for phenotypes, then some kind of mapping strategy is used to determine which mutation(s) cause the phenotype. The previous heyday of unbiased forward genetics in bacteria and yeasts led to discovery of functions of essential and non-essential genes, genes not yet annotated, and many useful kinds of non-null alleles that illuminated functions of proteins and pathways. These non-null alleles include altered-function alleles, conditional alleles, mutations in regulatory elements, and gain-of-function alleles that are particularly valuable in analysis of epistatic relationships (12). All of these types of mutations have played paramount roles in genetic discovery and dissection of gene and biological functions [e.g. (13–17)], organization (18) and regulation (19) of many paradigm-defining systems.

In unbiased forward-genetic strategies using chemical mutagenesis, the mapping step is the laborious, rate-limiting step, even at present with whole-exome sequencing and WGS readily available. On the one hand, the power of unbiased forward genetics and ‘mapping-by-sequencing’ is currently well appreciated in mouse (20), zebrafish and other model organisms (21) that are far more difficult and slow to manipulate than microbes. A NGS-driven renaissance of unbiased forward-genetic screens in these organisms is driving discovery of large, important gene networks and biological functions inaccessible otherwise, via screens that often take a year or more from mutagenesis to gene identification (20,21). The products of these screens are sufficiently valuable to justify the time and effort (20,21). On the other hand, surprisingly, the labor and difficulty of mapping even after WGS is such that modern methods in microbial genetics/genomics have virtually abandoned unbiased forward-genetic screens (using point mutagenesis and mapping) in even the most genetically tractable microbes, *E. coli*, other bacteria and yeasts.

In microbial model organisms, modern screen resources such as tagged libraries of selectable or barcoded deletion/replacements of non-essential genes (22–26), or transposon insertions (27–29) are typically employed in screens because of the ease of mapping the causative mutations after genetic screens. Knock-out libraries also provide 100% coverage of known non-essential protein-coding genes. However, essential genes occupy significant fractions of genomes such that knock-out libraries exclude many genes, and sometimes the key functions of interest. For example, *E. coli* has about 4500 genes (30) between 993 (22%) (31) and ∼300 (7%) (32) of which are estimated to be essential. Because screens in microbes can query tens of thousands of mutants, screens after random mutagenesis can provide deeper coverage than screens with deletion collections, which exclude essential genes, regulatory elements, genes not yet annotated, altered-function and other allele types. For example, a recent screen of random transposon-insertion mutants (not unbiased but less biased than knock-out collections) screened 69,000 mutants with estimated 94% saturation predicted by the numbers of multiple hits of genes, and identified several essential genes (33). By contrast screens of knock-out libraries exclude information from essential genes, genes not yet annotated, altered-function alleles, conditional alleles, and regulatory elements and the many important allele classes discussed above.

Although WGS should, in principle, allow identification of any mutation after screening, identifying the causative mutation after WGS is now the rate-limiting step in unbiased forward ‘genomic’ screens (forward genetics followed by WGS or NGS). Mutagenic treatments (or spontaneous mutagenesis) that give adequate yields of point mutants with the screened phenotype produce multiple mutations per genome. Determining which is causative after WGS (deconvolution) using traditional mapping methods is laborious and difficult, with the following exceptions.

First, mutations/mutant phenotypes that can be *selected* based on growth advantage from mixed populations, rather than *screened* (for any phenotype) in individual clones are more manageable. A recent elegant deconvolution method in *E. coli* selects for growth advantage (34). But this and other selections, such as for anti-microbial drug-resistance (35), cannot be used for most screened phenotypes.

Second, computational methods can find causative untagged mutations as multiple ‘hits’ among many sequenced mutants (36–39). But this works well only when very few genes—very small networks of about 10 or fewer—convey the particular phenotype. Higher numbers of phenotype-conferring genes (larger networks) reduce the frequency of occurrence of relevant mutations in pools of mutants (36–39). By contrast, bacterial [e.g. (33,40–43)] and yeast (44–47) gene networks routinely discovered even in biased forward-genetic screens of deletion collections and transposon-insertion mutants are often far larger, including many tens or hundreds of genes. These networks are expected to be even larger when unbiased strategies of chemical mutagenesis, and forward-genetic screens followed by sequencing are used (unbiased ‘forward genomics’).

Thus, despite the well appreciated power of unbiased forward-genomic screens, and the small genomes and tractability of microbes, currently, unbiased forward genomics is largely unused in the most genetically tractable microbial models such as *E. coli*. For example, as we write, a search for screens of chemically induced point mutants in *E. coli* shows the most recent to have been published twelve years ago (48). A rapid, general, and easy post-WGS mapping method has been lacking.

Here, we introduce ‘Deconvoluter’: a resource and method for rapid, precise and easy deconvolution of phenotype-causing screenable mutations in *E. coli* after WGS. The method takes mere days from WGS to identification of causative mutations, and is limited only by the ability to screen them. The Deconvoluter resource consists of ultra-dense libraries of intergenic insertions of selectable markers that allow rapid deconvolution of untagged causative mutations anywhere in the genome after WGS by a simple gene-replacement strategy. Singer *et al*. created the previous resource of this kind: an *E. coli* library of selectable transposon-insertion markers that can be used for mapping by replacement (49,50), which inspired the Deconvoluter strategy. The Deconvoluter libraries are much denser and are homogeneous (insertions spaced regularly) such that all genomic regions are covered. Thus, all possible point mutations anywhere in the genome can be deconvoluted rapidly and easily. We validate the libraries and method here with *N*-ethyl-*N*-nitrosourea (ENU)-induced mutations from a forward-genetic screen for altered fluorescence from a reporter gene. This resource makes unbiased forward-genetic screens with mapping-by-sequencing (‘forward genomics’) fast, simple and easy in *E. coli*. It additionally demonstrates a general strategy that could similarly enable rapid forward-genomic screens in many other microbes, both bacterial and eukaryotic.

## MATERIALS AND METHODS

### Strains and media

All bacterial strains used are derivatives of *E. coli* K12 strain MG1655 (51). Those mutagenized, sequenced and subsequently transduced also carry an mCherry-substituted derivative of the *gfp* gene in the Δ*attλ*::P*_sulA_gfp* chromosomal cassette of (52). Cultures were grown from single colonies with aeration at 37°C in LBH (5 g/l NaCl, 5 g/l Bacto-Yeast extract, 10 g/l Bacto-Tryptone, pH 7.5). For plate-reader assays cultures were grown in M9 minimal medium (53) supplemented with 0.1% glucose and 10 μg/ml vitamin B1 (M9 glucose B1). Where indicated, kanamycin was used at 30 μg/ml.

### Library preparation, whole-genome sequencing, read mapping and detection of genomic variants

Genomic (g) DNA was prepared from bacterial cultures using the Qiagen whole-genome prep kit. *Escherichia coli* genomic DNA (3 μg) was fragmented to an average size of 165 bp using a Covaris S2 sonicator (duty cycle 10%; intensity 5; 100 cycles/burst; 5°C; 6 min). Barcoded SOLiD 5500xl-compatible libraries were made from the fragmented gDNA using a SOLiD Fragment Library kit (Life Technologies) using standard manufacturer's protocols. Completed libraries were quantified by qPCR using a KAPA library quantification kit for Life Technologies SOLiD platform and pooled in equimolar quantities. The pooled libraries were sequenced on a SOLiD 5500xl sequencer to give paired-end (50 bp forward; 35 bp reverse) colorspace reads.

After determining the SOLiD read quality score metrics from each sequenced library, read alignment and variant calling with read data were conducted using the LifeScope 2.5.1 genomic analysis software (Life Technologies) for paired-end genomic reads. Briefly, reads were first processed using the SOLiD Accuracy Enhancement Tool (SAET) calibrated to the genome size of *E. coli*. Reads were then mapped to the *E. coli* reference genome (NCBI RefSeq: NC_000913.2 modified to contain the mCherry insert) using the gapped aligner and the subsequent alignments recorded in BAM format. Duplicate reads were then identified within the resulting BAM files and marked for filtering from downstream analysis.

Putative small insertion/deletion (indel) variants (up to 500 bp for deletions and 20 bp for insertions) were identified using the LifeScope small indel algorithm by processing gapped read alignment data. Putative single nucleotide variants (SNVs) were identified from the alignment data using LifeScope diBayes algorithm with parameters selected for low false-positive variant calls.

From primary read alignments a minimum base quality value of 20 was required for individual read bases to be considered in determining SNVs. Additionally, the SNV had to be identified from reads mapped to both strands of the reference sequence. For SNVs identified within the genomic coordinates of coding genes (based upon RefSeq genome annotation), potential changes to the amino acid translation of the gene were assessed by incorporating each variant into the gene nucleotide sequence and comparing the translated product to that of the unchanged gene.

Some SNVs were not detected with the above method. For some strains we called additional SNVs as follows: reads were mapped to the genome sequence of our reporter strain, the variant call was performed using a combination of variant calling with the SNVer tool (54) and SAMtools (55). The annotation was performed using the reporter strain genome.

### Assembly of the KO-Deconvoluter ultra-dense insertion library

We chose a subset of 94 deletion/insertion strains from the *E. coli* Keio deletion collection (22), which each have a non-essential gene replaced with a selectable kanamycin-resistance (Kan) cassette, with the Kan cassettes spaced every ∼50 kb in the *E. coli* genome (Supplementary Table S1). We used P1vir to generate an ordered library of phage P1vir stocks grown on each of the strains with the Kan cassettes chosen into our fluorescence reporter strain and tested them for being phenotypically silent with our fluorescence assay. All were silent.

### Construction of the i-Deconvoluter ultra-dense intergenic insertion library

This library has similar ∼50 kb spaced Kan cassettes in 94 strains (Table 1). To create this library, the FRT-Kan-FRT cassettes, with Kan gene flanked by FRT recombination sites for subsequent Kan removal with FLP recombinase if desired [per (56)], were amplified by PCR from pKD13 (56) and placed by phage lambda Red-mediated recombineering (56) into intergenic regions. Successful recombinants were selected on LBH kanamycin agar plates. Insertions were verified by PCR with specific primers (Supplementary Table S2). The Kan cassettes were transduced into the MG1655-derived reporter strain and tested for being phenotypically silent in the fluorescence assay. All were silent. We prepared an ordered library of phage P1vir stocks grown on each of the i-Deconvoluter strains.

**Table 1.** i-Deconvoluter library of *Escherichia coli* K12 strains

Library insertion number and allele name	Strain name	Insertion position (bp)^a^	Library insertion number and allele name	Strain name	Insertion position (bp)^a^
i-1	SMR20792	9245–9246	i-48	SMR20886	2 347 613–2 347 614
i-2	SMR20794	58 336–58 337	i-49	SMR20888	2 409 420–2 409 421
i-3	SMR20796	107 604–107 605	i-50	SMR20890	2 447 214–2 447 215
i-4	SMR20798	149 702–149 703	i-51	SMR20892	2 496 552–2 496 553
i-5	SMR20800	212 276–212 277	i-52	SMR20894	2 547 617–2 547 618
i-6	SMR20802	254 217–254 218	i-53	SMR20896	2 599 009–2 599 010
i-7	SMR20804	302 976–302 977	i-54	SMR20898	2 652 991–2 652 992
i-8	SMR20806	346 027–346 028	i-55	SMR20900	2 697 984–2 697 985
i-9	SMR20808	399 036–399 037	i-56	SMR20902	2 755 544–2 755 545
i-10	SMR20810	453 467–453 468	i-57	SMR20904	2 797 657–2 797 658
i-11	SMR20812	502 539–502 540	i-58	SMR20906	2 854 923–2 854 924
i-12	SMR20814	552 413–552 414	i-59	SMR20908	2 902 735–2 902 736
i-13	SMR20816	603 977–603 978	i-60	SMR20910	2 944 080–2 944 081
i-14	SMR20818	656 768–656 769	i-61	SMR20912	3 002 010–3 002 011
i-15	SMR20820	698 614–698 615	i-62	SMR20914	3 044 072–3 044 073
i-16	SMR20822	752 277–752 278	i-63	SMR20916	3 098 854–3 098 855
i-17	SMR20824	802 569–802 570	i-64	SMR20918	3 152 266–3 152 267
i-18	SMR20826	855 083–855 084	i-65	SMR20920	3 201 326–3 201 327
i-19	SMR20828	903 124–903 125	i-66	SMR20922	3 250 225–3 250 226
i-20	SMR20830	949 500–949 501	i-67	SMR20924	3 302 499–3 302 500
i-21	SMR20832	1 003 907–1 003 908	i-68	SMR20926	3 352 483–3 352 484
i-22	SMR20834	1 050 923–1 050 924	i-69	SMR20928	3 402 513–3 402 514
i-23	SMR20836	1 100 041–1 100 042	i-70	SMR20930	3 446 325–3 446 326
i-24	SMR20838	1 145 132–1 145 133	i-71	SMR20932	3 497 691–3 497 692
i-25	SMR20840	1 200 628–1 200 629	i-72	SMR20934	3 544 315–3 544 316
i-26	SMR20842	1 252 258–1 252 259	i-73	SMR20936	3 596 557–3 596 558
i-27	SMR20844	1 298 520–1 298 521	i-74	SMR20938	3 650 163–3 650 164
i-28	SMR20846	1 349 386–1 349 387	i-75	SMR20940	3 699 873–3 699 874
i-29	SMR20848	1 395 674–1 395 675	i-76	SMR20942	3 749 048–3 749 049
i-30	SMR20850	1 444 273–1 444 274	i-77	SMR20944	3 796 247–3 796 248
i-31	SMR20852	1 504 156–1 504 157	i-78	SMR20946	3 854 907–3 854 908
i-32	SMR20854	1 550 828–1 550 829	i-79	SMR20948	3 895 474–3 895 475
i-33	SMR20856	1 596 574–1 596 575	i-80	SMR20950	3 946 459–3 946 474
i-34	SMR20858	1 650 742–1 650 743	i-81	SMR20952	4 000 422–4 000 423
i-35	SMR20860	1 702 482–1 702 483	i-82	SMR20954	4 049 981–4 049 982
i-36	SMR20862	1 752 905–1 752 906	i-83	SMR20956	4 099 532–4 099 533
i-37	SMR20864	1 803 323–1 803 324	i-84	SMR20958	4 148 404–4 148 405
i-38	SMR20866	1 850 580–1 850 581	i-85	SMR20960	4 194 294–4 194 295
i-39	SMR20868	1 903 364–1 903 365	i-86	SMR20962	4 250 357–4 250 358
i-40	SMR20870	1 950 249–1 950 250	i-87	SMR20964	4 302 573–4 302 574
i-41	SMR20872	1 994 102–1 994 103	i-88	SMR20966	4 351 150–4 351 151
i-42	SMR20874	2 050 259–2 050 260	i-89	SMR20968	4 402 627–4 402 628
i-43	SMR20876	2 099 408–2 099 409	i-90	SMR20970	4 455 325–4 455 326
i-44	SMR20878	2 149 686–2 149 687	i-91	SMR20972	4 499 917–4 499 918
i-45	SMR20880	2 194 391–2 194 392	i-92	SMR20974	4 553 444–4 553 445
i-46	SMR20882	2 246 708–2 246 709	i-93	SMR20976	4 597 553–4 597 569
i-47	SMR20884	2 306 675–2 306 676	i-94	SMR20978	4 626 644–4 626 645

^a^Reference for position is *E. coli* K12 sequenced reference strain MG1655 (65), the strain background used to house this library.

### P1vir stock preparation

Phage P1vir is used widely for generalized transduction of *E. coli* genome segments replacing the homologous segment in the recipient of transduction with sequence from the ‘donor’ *E. coli* strain on which the phage were grown (53). To prepare transducing stocks, each single donor-strain colony was grown overnight in LBH liquid medium with 30 μg/ml kanamycin in a 14 ml polypropylene round-bottom tube with vigorous aeration at 37°C. Saturated cultures were diluted 1:100 into 4 ml LBH with 5 mM CaCl_2_, 0.4% glucose and incubated for 1 h at 37°C without shaking. Cultures were then infected with ∼3 × 10^6^ pfu of P1vir (previously grown on stain MG1655) and incubated for an additional 2–3 h shaking at 37°C. When cultures were cleared by apparent lysis, 100 μl of CHCl_3_ were added per tube and the tubes vortexed three times for 30 s. Debris were pelleted (SLA-1500, 7000 rpm, 7 min, 4°C) and supernatant suspensions of phage P1 particles (P1 stocks) were collected into sterile screw-cap glass tubes and stored at 4°C.

### High-throughput deconvolution of fluorescence phenotypes in a multi-well plate reader

ENU-mutagenized strains (53) were grown overnight in LBH, in 14 ml polypropylene round-bottom tubes shaking at 37°C. The saturated cultures were divided into 100 μl aliquots, one for each SNV to be tested in each strain. Each aliquot was mixed with 500 μl salt solution (15 mM CaCl_2_, 30 mM MgSO_4_) and 100 μl of the corresponding P1vir stock from the Deconvoluter libraries, and left shaking for 20 min at 37°C. Afterwards 4.5 ml of LBH with 20 mM sodium citrate were added and cells pelleted (SLA-1500, 7000 rpm, 7 min, RT), then resuspended in 300 μl LBH with 20 mM sodium citrate and incubated shaking for 30 min at 37°C to express kanamycin resistance, then pelleted again, resuspended in LBH (+ 20 mM sodium citrate with kanamycin) and either plated directly onto LBH/kanamycin-citrate OmniTray plates (Thermo Fisher Scientific, Rochester, NY, USA) (recommended for obtaining independent transductants), or were grown for ∼8 h to OD_600_ of 0.2, then diluted 1:100 in M9 salts (53) and 100 μl were then plated (not recommended). The plates were incubated overnight at 37°C.

Handling of the plates was done in a RapidPick Workcell robot (Hudson Robotics, Springfield, NJ, USA). From each plate, 22 transductants were picked into a 384-well plate (μ-clear, black, Greiner Bio-One, Monroe, NC, USA) containing 80 μl M9 glucose B1 medium per well. Plates were sealed with a breathable membrane and incubated shaking overnight at 37°C. The next day the membranes were removed and the plates analyzed in a Synergy 2 fluorescence plate reader (BioTek, Winooski, VT, USA). Fluorescence phenotypes were scored as relative fluorescence per OD_600_ unit.

### Predicted co-transduction with known *E. coli* genes

Co-transduction frequencies were predicted using the formula of Wu (57), based on the midpoint of genes as annotated in EcoGene 3.0 (58) downloaded in Jan 2015. Coordinates from Table 1 and Supplementary Table S1 were corrected for differences between U00096.2 and U00096.3 coordinates and distances were calculated using a transducing segment size of 100 kb for phage P1. For the KO-Deconvoluter alleles, distances were taken from the nearest edge of the deletion-replacement.

### Data access

An enhanced searchable version of the linkage calculations for the Deconvoluter libraries (Supplementary Table S3) are available online at PortEco EcoliWiki (59) (http://ecoliwiki.net/tools/deconvoluter/). The positions of the two Deconvoluter library members can also be viewed in genome context using the Deconvoluter track at http://browser.porteco.org.

## RESULTS AND DISCUSSION

We constructed Deconvoluter: ultra-dense libraries of selectable intergenic insertions that allow rapid deconvolution of untagged causative mutations after WGS in *E. coli*. We developed two ordered, ultra-dense insertion libraries. Both allow rapid deconvolution of unselectable (but screenable), untagged mutations after forward-genetic screening and WGS. Each library has a selectable marker spaced every 50 kb in the *E. coli* genome. These are used to replace sequenced point (or other small) mutations, for example in chemically-induced mutants, with wild-type sequence near the selectable insertion. This allows determination of which sequenced mutation is causative by reversion of the screened phenotype. The Deconvoluter libraries can also be used to identify causative genomic regions when WGS has missed the causative single-nucleotide variant (SNV), or in unsequenced strains.

### Forward-genomic strategy

**Figure 1.**
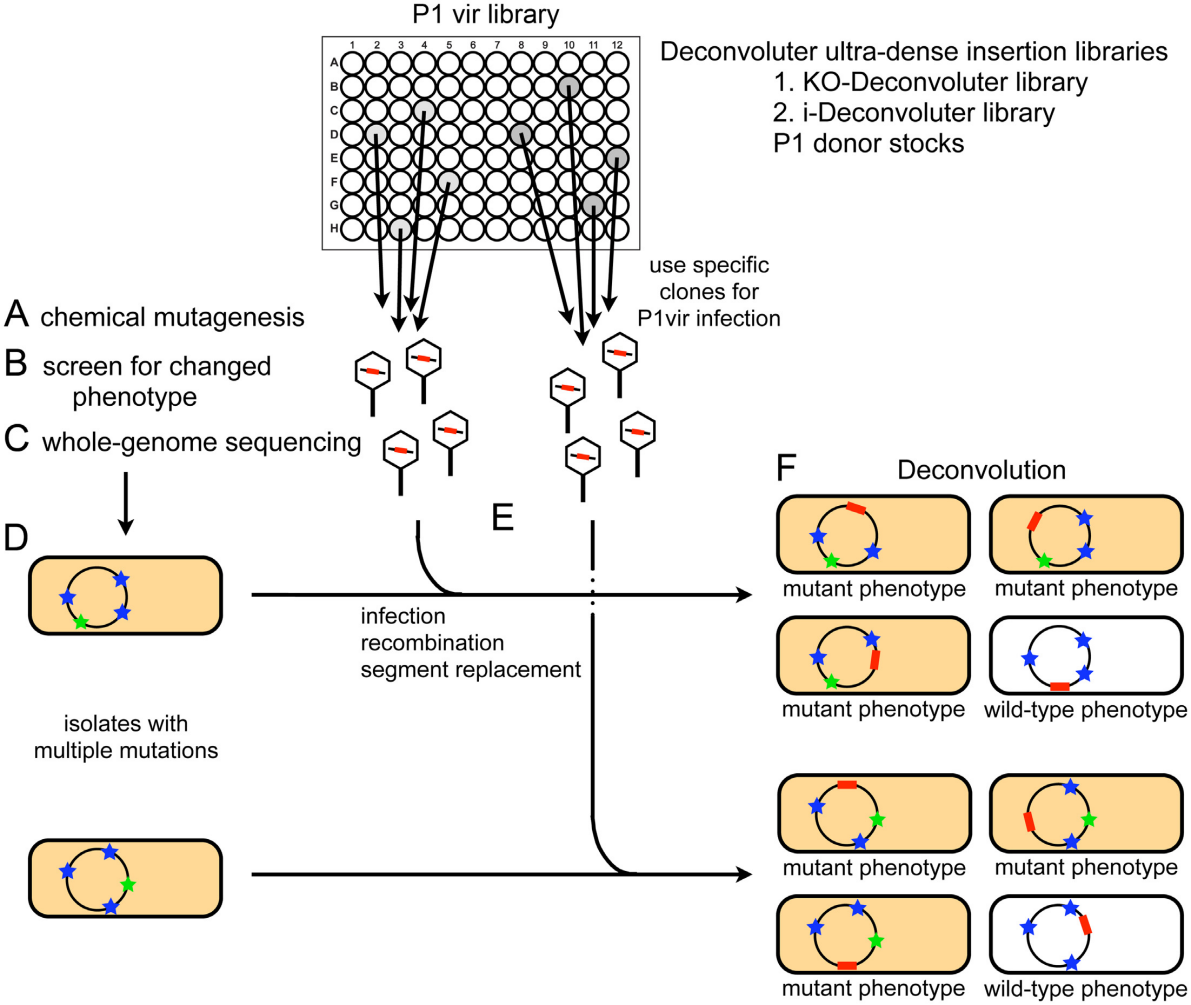
Strategy for discovery of causative mutations from sequenced genomes using Deconvoluter ultra-dense insertion libraries. (**A**–**D**) *E. coli* are treated with a chemical mutagen to induce random point mutations (stars) in the genome (circles) including non-null alleles of essential genes. (**B**) After screening for changes in the phenotype (orange cells in **D** and **F**) and (**C**) whole-genome sequencing (WGS), (**D**) the not-yet-identified causative mutation (green star) is deconvoluted from the multiple non-causative mutations (blue stars) via a simple gene-replacement strategy using the Deconvoluter libraries. Circles, 4.6 MB *E. coli* chromosome. (**E**) Deconvolution by phage P1-based transductional gene replacement with library of P1 phage grown on Deconvoluter ultra-dense selectable insertion libraries, followed by (**F**) testing for reversion of the screened mutant phenotype (depicted as reversion to white cells). The two Deconvoluter *E*.*coli*strain libraries on which P1 is grown carry selectable kanamycin-resistance markers (red bars), spaced every ∼50 kb in the genome. The i-Deconvoluter library (Table 1) has 94 intergenic insertions. The KO-Deconvoluter library (Supplementary Table S1) carries 94 selectable gene deletions. After picking the transductants (recombinants) from antibiotic-containing medium, the isolates are re-screened to test whether the phenotype-causing mutation has been reverted (white cells).

Deconvolution with the Deconvoluter ultra-dense insertion libraries works as follows (Figure 1). A population of *E. coli* is mutagenized with a chemical mutagen to produce several mutations per genome, then subjected to a genetic screen for a phenotype of interest (symbolized as orange-colored cells, Figure 1D and F). In our test case, we aimed for ∼6 mutations per genome. Following mutagenesis, mutants may be identified by any screening procedure, including for altered colony color or morphology, or, as we did, by altered fluorescence from a reporter gene assayed in a multi-well plate reader. WGS of the mutants from unbiased screens identifies multiple mutations per genome, most of which are unrelated to the screened phenotype (blue stars, Figure 1D) and usually only one or few of which causes the screened phenotype (green stars, Figure 1D). To deconvolute the causative mutation from the several non-causative mutations, each sequenced mutation is replaced separately by wild-type sequence linked with a nearby neutral selectable marker—an antibiotic-resistance gene cassette (red bars, Figure 1E and F)—from one of the two Deconvoluter libraries. Replacement with the wild-type sequence that flanks the cassette is executed using a simple transduction protocol with phage P1vir. The transductants (recombinants) with each chemically-induced mutation replaced are then re-screened to see which no longer displays the mutant phenotype (reversion to white cells, Figure 1F), thus identifying which sequenced mutation was causative.

**Figure 2.**
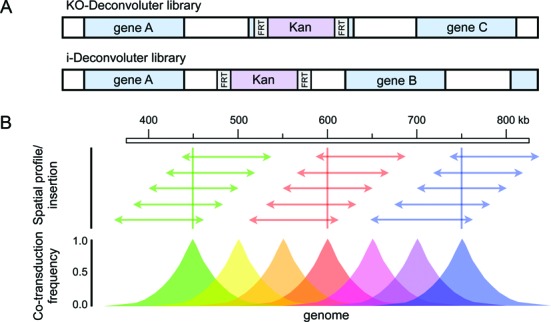
Deconvoluter libraries cover the *E. coli* genome via transduction-based gene-replacement. (**A**) Kan-insertion schemes of the KO-(deletion/replacement) and i-(intergenic insertion) Deconvoluter libraries. For the i-Deconvoluter library we engineered removable Kan cassettes into intergenic regions of the genome creating a library of 94 strains, each with a single intergenic insertion spaced 50 kb apart across the 4.6 MB *E. coli* genome (Table 1). The linkage of these alleles to all known genes in the *E. coli* genome is given in Supplementary Table S3. (**B**) Strategy for deconvolution of untagged mutations in multiply mutated genomes using phage P1 transductional replacement. Each set of colored arrows illustrates a continuous set of 100 kb genomic lengths that can be replaced by transduction and selection of each Kan insertion at each 50 kb-spaced Kan-insertion site. We illustrate arrays of wild-type DNA segments that can be co-transduced with Kan insertions at positions 450 (green), 600 (red) and 750 kb (blue), but similar arrays apply to each Kan at each position. The graph represents the idealized co-transductant frequency of each Kan with the wild-type sequence that would replace any putative mutation depending on the distance of the sequenced mutation from the Kan cassette [modified from (57)]. Calculated co-transduction efficiencies for all known *E. coli* genes with nearby insertions in both the KO- and i-Deconvoluter libraries are shown in Supplementary Table S3.

The maximum length of DNA replaced by homologous recombination during transduction by phage P1 is ∼100 kb. Thus, our libraries with selectable markers every 50 kb in the *E. coli* genome cover the whole genome (Figure 2B). Similarly, the libraries may be most useful for deconvolution of mutations smaller than 100 kb including base substitutions, indels, deletions, transposon and other insertions and rearrangements of <100 kb.

### Two donor libraries

Each of the two donor libraries includes 94 strains, each with a kanamycin-resistance (Kan) cassette inserted every ∼50 kb into the genome (Figure 2A and B, Table 1, Supplementary Table S1). Such dense coverage ensures that the co-transduction frequency to replace any sequenced mutation with wild-type sequence linked with the neutral Kan marker does not drop below ∼35% with respect to the nearest marker in the library (57) (Supplementary Table S3). The KO-Deconvoluter library (Figure 2A, Supplementary Table S1), includes 94 deletion/insertion strains of non-essential genes replaced with a selectable Kan cassette assembled from the *E. coli* Keio deletion collection (22). This library was useful for us in a screen of mutants that alter induction of the *E. coli* SOS DNA-damage response, because we showed that no donor strain of the KO-Deconvoluter library affects spontaneous SOS induction levels measured via flow cytometry with a modified version of the chromosomal SOS-fluorescence reporter of Pennington and Rosenberg (52). We engineered a second Deconvoluter library that is likely to be more useful for more screens for various phenotypes because no known gene is disrupted: the i-Deconvoluter library (Figure 2A, Table 1). To create the i-Deconvoluter library, we engineered a removable Kan cassette every ∼50 kb in the genome in intergenic regions: 40% between convergently transcribed genes; 49% between genes transcribed co-directionally, and 11% between divergently transcribed genes. The i-Deconvoluter library includes 94 strains, each with a single intergenic insertion. Although none is predicted to affect any known *E. coli* gene, any might possibly alter a gene or regulatory region not yet annotated, or affect expression of a neighboring gene. All strains in this library were found to be phenotypically neutral in our fluorescence assay. We suggest that, similarly, others may wish determine whether any insertion confers a phenotype that might limit its utility in particular screens of interest.

### Co-transduction with known genes

Supplementary Table S3 shows predicted co-transduction frequencies of the i-Deconvoluter and KO-Deconvoluter insertions with all known genes in the *E. coli* genome based on the formula of Wu (57).

### Deconvolution of ENU-induced mutations

As a test case, we induced mutations with *N*-ethyl-*N*-nitrosourea (ENU) in our reporter strain at a dose that gave ∼6 mutations/genome to ensure that most cells carry at least one mutation (Supplementary Figure S1). Probabilities of clones with 0, 1 and >1 mutations per genome at various levels of mutagenesis are given in Supplementary Table S4. We verified the dose-dependent mutation frequency by WGS of unscreened treated survivors of this regimen. We screened for increased or decreased fluorescence of a strain which reports fluorescence from a chromosomal reporter gene upregulated by the SOS DNA-damage response [modified from (52)]. Cells were treated with ENU, plated for single colonies; the mutants were picked by a colony-picking robot into 384-well plates and assayed for fluorescence (SOS response) relative to optical density (cell number). Mutants with altered fluorescence were isolated, purified, and sequenced.

**Figure 3.**
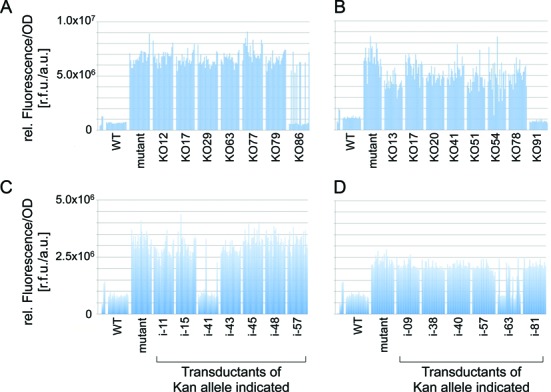
Deconvoluter libraries identify ENU-induced causative mutations using a fluorescence-based plate-reader screen. (**A**–**D**) Examples of deconvolution by phenotype reversion after transductional gene replacement. For each mutation identified by WGS the nearest Kan was transduced using P1 phage; 22 Kan-resistant colonies were picked robotically into 384-well plates, grown to saturation and analyzed by plate reader for fluorescence from a reporter gene: the initially screened phenotype. Each bar in each group shows the relative fluorescence for each of the 22 transductants of the named insertions. Reversion of the fluorescence phenotype among some of the transductants of a given Kan insertion identifies the causative mutation. Each panel represents a different ENU-mutant strain/isolate with the number of genomic mutations indicated by the number of KO- or i-Deconvoluter donor stocks used. This deconvolution method works with the KO-Deconvoluter library (**A**, **B**) as well as with the i-Deconvoluter library (**C**, **D**). The distances of the Kan elements from the mutations identified as causative in this set were 7, 16, 9 and 3 kb, respectively, for the mutations deconvoluted in (**A**)–(**D**), respectively.

We used generalized transducing phage P1vir (60) grown on either the KO-Deconvoluter or i-Deconvoluter library strains and generated ordered phage-suspension (phage ‘stock’) libraries from each Deconvoluter bacterial library. We chose phage stocks with the Kan marker nearest to each mutation identified by WGS, and transduced the Kan cassettes with linked wild-type sequence into the mutant strains in parallel. Using a robot (Hudson Robotics), for each transduction, we picked 22 Kan-resistant colonies into liquid medium arrayed in 384 well plates. After 18 hours of growth, fluorescence measurements using a plate reader allowed identification of the causative mutation as those transductions in which some of the transductants no longer had the increased-fluorescence phenotype (Figure 3A–D). As the data show, both the KO-Deconvoluter library (Figure 3A and B) and the i-Deconvoluter library (Figure 3C and D) deconvoluted the causative mutation from multiple genomic mutations. We analyzed 17 mutant strains, with a mean of 6 ± 3 SD (range: 2–14) mutations per genome (Figure 3, Supplementary Figure S2). We successfully identified causative mutations that account for the whole phenotype in 16 of the 17. In the remaining strain, a Kan cassette identified an apparent partially causative SNV/region in which our replacement with wild-type sequence partially restored wild-type phenotype, as if an additional uncalled mutation elsewhere also contributed to the phenotype (Supplementary Figure S2K). Modification of the phenotype of a mutation by other mutations (epistatic interactions) is also found in studies of cultures evolved over time (61). A few genomes showed no mutations, even though the mutant phenotype was displayed, indicating imperfectly efficient mutation detection in our WGS pipeline. However, the Deconvoluter method was highly efficient for identifying causative mutations in 17 of 17 tested.

### Predicted co-transduction and resolution of close mutations

The formula of Wu (57) for co-transduction predicts the probability of separation of close mutations via transduction with a Deconvoluter insertion. For two mutations that fall in the same 50 kb interval between Kan cassettes, the probability of their separation upon transduction increases with the distance between them per (57). Supplementary Table S5 shows examples of the probabilities of resolving two mutations separated by 1, 5 and 10 kb at varying distances from the Kan cassette. For example, mutations 10 kb apart and ≤30 kb from the Kan cassette can be separated with 17–22% probability; mutations 5 kb apart up to 25 kb from a Kan cassette can be resolved with ∼10% probability. For mutations 1 kb apart the probability of resolving the mutations by transduction drops to 2–3%.

### Rapidity of the method

The time from sequenced genome to phenotypic reversion and deconvolution with this method used with our fluorescence assay was three days. On day 1 after WGS-identification of all mutations in the genomes of mutants with phenotypes, the mutants were transduced separately with each of the P1 stocks with markers near to each sequenced mutation. On day 2, transductant colonies were picked robotically into 384-well plates and grown to saturation overnight. On day 3, the plates were assayed in a fluorescence plate reader, reverted-fluorescence phenotypes detected, and causative mutations identified. Three days from WGS to deconvolution compares very favorably with the year or more needed for deconvolution by ‘mapping-by-sequencing’ in mouse and zebra fish (20,21) and should, we believe, make this method highly desirable to the bacterial genetics community. If vertebrate geneticists can wait years for the benefits of chemical mutagenesis then mapping-by-sequencing, perhaps the resource presented here may persuade bacterial geneticists to exploit the depth of and power inherent in unbiased forward-genomic screens.

### General applicability

This strategy is also expected to serve as a general model for creation of similar libraries for many other microbial models, bacterial and eukaryotic, in which this strategy would be similarly likely to work. Most microbial models have tractable gene-replacement methods in which linked non-mutant DNA is co-replaced with a selectable marker (natural transformation, transduction, conjugation, linear gene replacement) and so could support the Deconvoluter method with similarly constructed libraries.

### Tagging mutations

An additional utility of Deconvoluter is that the method simultaneously creates Kan (selectable) cassette linkages to the causative chemically-induced mutation, which can be used for subsequent transduction of the causative mutations into any transducible *E. coli* strain desired. Because the selectable Kan cassettes of the Deconvoluter libraries are only partially linked with the untagged mutations, some transductions generate a Kan-linked mutant allele that has not reverted the phenotype, even though many with the same cassette have replaced the mutation and reverted the phenotype (Figure 3, the high-fluorescence isolates from transductions with insertions KO86, i-41, and i-63 in panels A, C and D, respectively). This marked allele of the causative mutation is then transduced into the wild-type strain for verification. Transduction of 16 identified causative mutations into the wild-type strain confirmed the phenotype of all of them.

## CONCLUSION

The Deconvoluter libraries allow simple, rapid, high-throughput deconvolution of multiple unmarked, unselectable mutations in *E. coli*. The Deconvoluter strategy allows the harnessing of highly economical WGS of organisms with small genomes for unbiased forward-genomic screens that allow ready detection of non-null essential-gene mutations, gain-of-function and altered-function alleles, conditional alleles, mutations in genes not yet annotated, and in both known and as-yet-undiscovered regulatory regions. Deconvoluter uses removable Kan cassettes [per (22)] that allow interrogation of multiple mutations sequentially.

The i-Deconvoluter library may be most generally useful in that no known gene is disrupted in it. It is possible that a gene deletion or even a presumed intergenic insertion cassette might have secondary side-effects when combined with a new chemically-induced mutation, or that an intergenic insertion may interfere with a currently undiscovered gene or regulatory element in a genomic region. In case of uncertainty about the causative effect of a mutation, the stocks of other nearby insertions from either library can be used to verify causative mutations independently. Supplementary Table S3 shows predicted co-transduction frequencies with known *E. coli* genes.

The high-throughput Deconvoluter method is a powerful approach for deconvoluting unselectable (but screenable) untagged mutations in unbiased forward-genomic screens. These libraries and this method will allow unbiased discovery of large gene networks that are inaccessible to current multiple-hit computational methods alone, and should assist in restoring the ability to use microbes for deep translation of basic molecular-biological discovery across phylogeny. Given their rich history in basic and applied molecular biological advances [reviewed, Introduction, and, e.g. (62–64)], rigorous experimental facility, rapid growth, economy, and small genome sizes, microbes are ideal for modern functional genomic, systems biological and synthetic applications.

## Supplementary Material

SUPPLEMENTARY DATA
