# Supplementary material for: An ultra-dense library resource for rapid deconvolution of mutations that cause phenotypes in Escherichia coli
Source: Nucleic Acids Res. 2015 Nov 17;44(5):e41. doi: 10.1093/nar/gkv1131 (PMC4797258; doi:10.1093/nar/gkv1131)
Supplement: SUPPLEMENTARY DATA [file supp_44_5_e41__index.html]

An ultra-dense library resource for rapid deconvolution of mutations that cause phenotypes in Escherichia coli — SUPPLEMENTARY DATA 

# An ultra-dense library resource for rapid deconvolution of mutations that cause phenotypes in *Escherichia coli*

## SUPPLEMENTARY DATA

- SUPPLEMENTARY DATA
- SUPPLEMENTARY DATA
- SUPPLEMENTARY DATA
- SUPPLEMENTARY DATA
- SUPPLEMENTARY DATA
- SUPPLEMENTARY DATA
- SUPPLEMENTARY DATA
- SUPPLEMENTARY DATA
