# Supplementary material for: An ultra-dense library resource for rapid deconvolution of mutations that cause phenotypes in Escherichia coli
Source: Nucleic Acids Res. 2015 Nov 17;44(5):e41. doi: 10.1093/nar/gkv1131 (PMC4797258; doi:10.1093/nar/gkv1131)
Supplement: SUPPLEMENTARY DATA [file supp_gkv1131_nar-01366-met-k-2015-File005.pdf]

SUPPLEMENTARY LEGENDS

Supplementary Table S1. KO-Deconvoluter library of *Escherichia coli* K12 strains.
Supplementary Table S2. Primers for PCR confirmation of Kan insertions.
Supplementary Table S3. Predicted co-transduction frequencies of known genes with Deconvoluter insertions.
Supplementary Table S4. Probabilities of occurrence of zero, one, and more than one mutation per genome at varying levels of mutagenesis.
Supplementary Table S5. Probabilities of independent transduction of two neighboring mutations.
Supplementary Figure S1. Dose-response curve of forward rifampicin-resistance mutations and mutations per genome obtained with varying doses of ENU.
Supplementary Figure S2. Additional examples of ENU-induced causative mutations identified using Deconvoluter libraries and a fluorescence-based plate-reader screen.
