# Supplementary material for: An ultra-dense library resource for rapid deconvolution of mutations that cause phenotypes in Escherichia coli
Source: Nucleic Acids Res. 2015 Nov 17;44(5):e41. doi: 10.1093/nar/gkv1131 (PMC4797258; doi:10.1093/nar/gkv1131)
Supplement: SUPPLEMENTARY DATA [file supp_gkv1131_nar-01366-met-k-2015-File006.pdf]

A

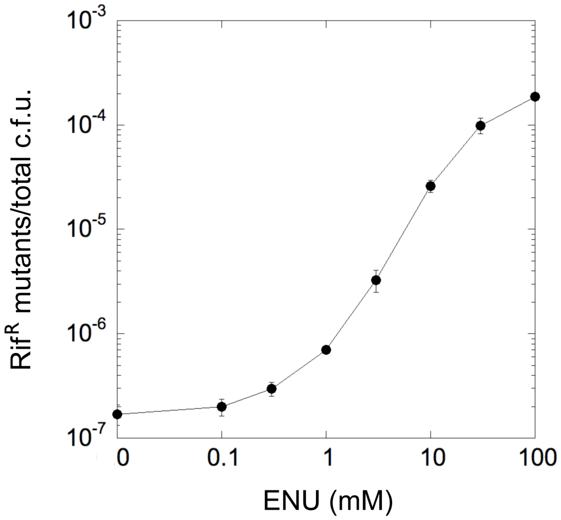

B

ENU (mM)	0	1	10	30	100
n=	1	1	5	5	2
Mut./Genome	1	1	6±2	19±12	33±2

Supplementary Figure S1. Dose-response curve of forward rifampicin-resistance mutations and mutations per genome obtained with varying doses of ENU. (**A**) The *E. coli* reporter strain was treated with increasing doses of ENU to induce point mutations and plated on rifampicin plates to estimate the frequency of rifampicin-resistant (Rif^R) forward mutations in the *rpoB* gene. Values are the average of five experiments ± SEM. (**B**) WGS of the number of isolates shown (n) revealed the numbers of mutations per genome indicated.
