# Supplementary material for: An ultra-dense library resource for rapid deconvolution of mutations that cause phenotypes in Escherichia coli
Source: Nucleic Acids Res. 2015 Nov 17;44(5):e41. doi: 10.1093/nar/gkv1131 (PMC4797258; doi:10.1093/nar/gkv1131)
Supplement: SUPPLEMENTARY DATA [file supp_gkv1131_nar-01366-met-k-2015-File007.pdf]

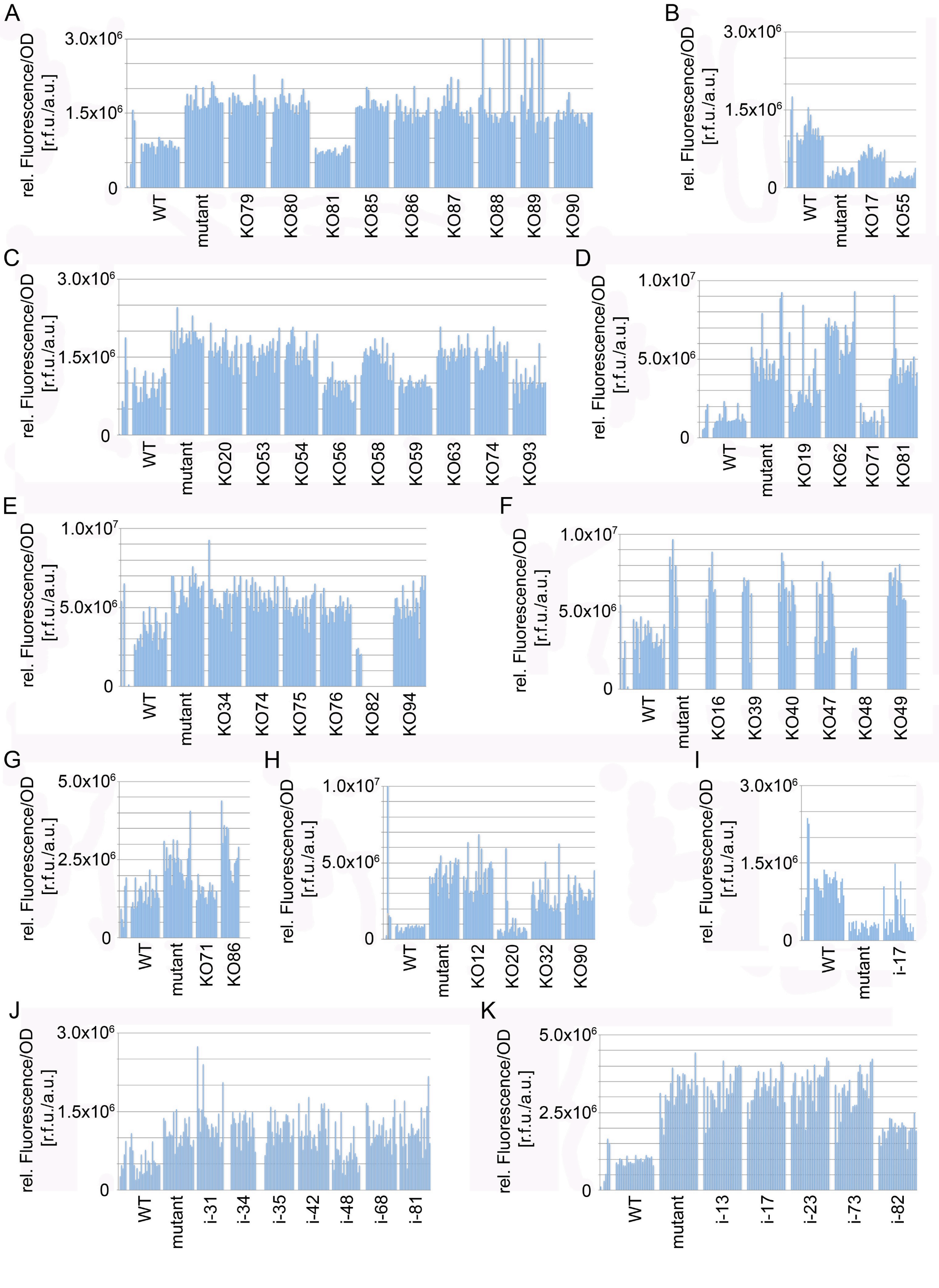
A
rel. Fluorescence/OD [r.f.u./a.u.]
3.0x10^6
1.5x10^6
0
WT mutant KO79 KO80 KO81 KO85 KO86 KO87 KO88 KO89 KO90
B
rel. Fluorescence/OD [r.f.u./a.u.]
3.0x10^6
1.5x10^6
0
WT mutant KO17 KO55
C
rel. Fluorescence/OD [r.f.u./a.u.]
3.0x10^6
1.5x10^6
0
WT mutant KO20 KO53 KO54 KO56 KO58 KO59 KO63 KO74 KO93
D
rel. Fluorescence/OD [r.f.u./a.u.]
1.0x10^7
5.0x10^6
0
WT mutant KO19 KO62 KO71 KO81
E
rel. Fluorescence/OD [r.f.u./a.u.]
1.0x10^7
5.0x10^6
0
WT mutant KO34 KO74 KO75 KO76 KO82 KO94
F
rel. Fluorescence/OD [r.f.u./a.u.]
1.0x10^7
5.0x10^6
0
WT mutant KO16 KO39 KO40 KO47 KO48 KO49
G
rel. Fluorescence/OD [r.f.u./a.u.]
5.0x10^6
2.5x10^6
0
WT mutant KO71 KO86
H
rel. Fluorescence/OD [r.f.u./a.u.]
1.0x10^7
5.0x10^6
0
WT mutant KO12 KO20 KO32 KO90
I
rel. Fluorescence/OD [r.f.u./a.u.]
3.0x10^6
1.5x10^6
0
WT mutant i-17
J
rel. Fluorescence/OD [r.f.u./a.u.]
3.0x10^6
1.5x10^6
0
WT mutant i-31 i-34 i-35 i-42 i-48 i-68 i-81
K
rel. Fluorescence/OD [r.f.u./a.u.]
5.0x10^6
2.5x10^6
0
WT mutant i-13 i-17 i-23 i-73 i-82

L

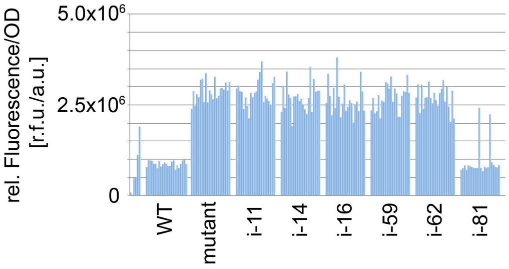

M

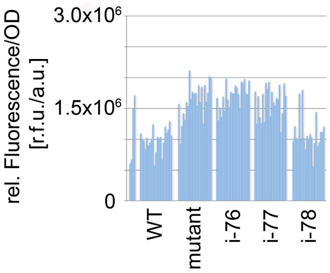

Supplementary Figure S2. Additional examples of ENU-induced causative mutations identified using Deconvoluter libraries and a fluorescence-based plate-reader screen. See the Figure 3 legend for details. Each panel represents a different ENU-mutant strain deconvoluted by the KO-Deconvoluter library (**A**-**H**) or the i-Deconvoluter library (**I-M**). The number of causative mutations discovered (and verified by crossing out and re-testing) and insertion that was partially linked with it are as follows: (**A**) one, KO81; (**B**) one, KO17; (**C**) three, of which one linked with KO56 conferred the phenotype when transferred into a non-mutated strain. The other two may be mutations that confer the phenotype only in the presence of another mutation(s) in that genome; (**D**) one, linked with KO71, confers the phenotype when transferred to a mutation-free strain. A possible other mutation linked with KO19 might also be causative either in the presence of other mutations in this strain or not (not tested). (**E**) one, KO82; (**F**) one, KO48; (**G**) one, KO71; (**H**) one, KO20; (**I**) one, i-17; (**J**) one, i-48; (**K**) one, i-82; (**L**) one, i-81; (**M**) one, i-78.
