# Supplementary material for: An ultra-dense library resource for rapid deconvolution of mutations that cause phenotypes in Escherichia coli
Source: Nucleic Acids Res. 2015 Nov 17;44(5):e41. doi: 10.1093/nar/gkv1131 (PMC4797258; doi:10.1093/nar/gkv1131)
Supplement: SUPPLEMENTARY DATA [file supp_gkv1131_nar-01366-met-k-2015-File011.pdf]

Supplementary Table S4. Probabilities of occurrence of zero, one, and more than one mutation per genome at varying levels of mutagenesis

Mean # mutations/genome	Probability P0 0 mutation/genome	Probability P1 1 mutation/genome	Cumulative probability P>1 >1 mutation/genome	Main expense
6	0.002	0.015	0.983	Deconvolution
1	0.368	0.368	0.264	Sequencing
0.5	0.607	0.303	0.090	Screening
0.25	0.779	0.195	0.026	Screening
0.2	0.819	0.164	0.017	Screening
0.1	0.905	0.090	0.005	Screening
0.01	0.990	0.010	0.000	Screening

The probabilities of various numbers of mutations per genome can be estimated by a Poisson calculation to project the cost / burden of a screen at varying levels of mutagenesis. This can be compared with the cost of mutagenesis at low doses and WGS without deconvolution, using data from mutants with one mutation per genome. For example, for our test-case screen we chose 10 mM ENU for chemical mutagenesis to generate an average of 6 mutations per genome (see Supplementary Figure S1), which results in most cells having at least one mutation, thus keeping the total number of clones to be screened low (less costly). The table shows that under these circumstances about 98% of the screened cultures have more than one mutation and the number of non-mutated colonies picked, grown into liquid cultures and screened is negligible. With these conditions, identifying causative mutations requires deconvolution with the libraries as described here. Selecting a chemical dose to generate an average of one mutation per genome will generate about 37% of “dead volume” in the screen—colonies with no mutation—and still produce 26% of colonies with more than one mutation. This 26% portion will be found in the phenotypic screen, validated and whole-genome sequenced. After sequencing these isolates would be discarded if neither our libraries nor previous, more laborious methods were used for deconvolution. Reducing the mutation load to a mean of one mutation per two genomes (a frequency at which only ~10% of the sequenced strains have to be discarded) would increase the number of clones to be screened approximately 300-fold above the conditions we used aiming for mean of 6 mutations per genome.
