# Supplementary material for: An ultra-dense library resource for rapid deconvolution of mutations that cause phenotypes in Escherichia coli
Source: Nucleic Acids Res. 2015 Nov 17;44(5):e41. doi: 10.1093/nar/gkv1131 (PMC4797258; doi:10.1093/nar/gkv1131)
Supplement: SUPPLEMENTARY DATA [file supp_gkv1131_nar-01366-met-k-2015-File012.pdf]

Supplementary Table S5. Probabilities of independent transduction of two neighboring mutations

2 Mutations, 1kb apart			2 Mutations, 5kb apart			2 Mutations, 10kb apart		
Distance from Kan cassette (kb)	Co-transductant frequencies with Kan cassette*	Probability of independent transduction**	Distance from Kan cassette (kb)	Co-transductant frequencies with Kan cassette*	Probability of independent transduction**	Distance from Kan cassette (kb)	Co-transductant frequencies with Kan cassette*	Probability of independent transduction**
10<br>11	0.729<br>0.705	0.024	10<br>15	0.729<br>0.614	0.115	10<br>20	0.729<br>0.512	0.217
20<br>21	0.512<br>0.493	0.019	20<br>25	0.512<br>0.422	0.090	20<br>30	0.512<br>0.343	0.169
30<br>31	0.343<br>0.329	0.014	30<br>35	0.343<br>0.275	0.068	30<br>40	0.343<br>0.216	0.127
50<br>51	0.125<br>0.118	0.007	50<br>55	0.125<br>0.091	0.034	40<br>50	0.216<br>0.125	0.091

* = Co-transductant frequencies calculated per Wu: f = $[1 - (\text{distance between marker position and mutation/length of transduced DNA})]^3$
** = Probability of independent transduction is the difference between the co-transduction frequencies

There is some ability to separate mutations closer than the distance between Deconvoluter Kan-insertion markers. For two mutations that fall in the same 50kb interval, the probability of their independent transduction can be calculated by the Wu formula (57), which allows calculation of the expected co-transduction frequency of two markers. The Table shows examples of such calculations for two mutations that are 1kb, 5kb and 10 kb apart for varying distances from the Kan cassette. Mutations 10kb apart ≤30kb from the Kan cassette can be separated with 17-22% probability; mutations 5kb apart up to 25kb from a Kan cassette can be resolved with ~10% probability. For mutations 1kb apart the probability of resolving the mutations by transduction drops to 2-3%.
